# Polyethylene Packaging as a Source of Microplastics: Current Knowledge and Future Directions on Food Contamination

**DOI:** 10.3390/foods14142408

**Published:** 2025-07-08

**Authors:** Piotr Kowalczyk, Kornelia Kadac-Czapska, Małgorzata Grembecka

**Affiliations:** Department of Bromatology, Faculty of Pharmacy, Medical University of Gdansk, 80-416 Gdańsk, Poland; piotr.kowalczyk50668@gumed.edu.pl (P.K.); kornelia.kadac-czapska@gumed.edu.pl (K.K.-C.)

**Keywords:** polyethylene, microplastic, degradation, adsorption, bioaccessibility, toxicity, food packaging

## Abstract

Polyethylene (PE) is the most widely produced plastic globally. It is extensively used as packaging in both the food and pharmaceutical industries. Its use can result in the formation of emerging contaminants—microplastics (MPs). This review summarizes current knowledge on PE and PE-derived microplastics (PE–MPs) and highlights existing gaps. It discusses the factors influencing PE degradation, with particular emphasis on interactions with packaged contents and food products. The role of PE–MPs as vectors for environmental contaminants is also examined, focusing on their adsorption and desorption behavior. Finally, we explore the toxicity and bioaccessibility of PE–MPs. Our findings indicate that pH, temperature, and exposure time are the most significant factors driving PE degradation. However, comparative studies examining a broad spectrum of parameter values remain scarce. The process of PE–MP generation remains largely unexplored. Adsorption mechanisms on PE–MPs are well documented in the literature. In contrast, desorption has received significantly less scientific attention, and its relevance to human exposure is still unclear. Numerous studies have suggested potential links between human exposure to PE–MPs and the development of non-communicable diseases, including cardiovascular and neurodegenerative disorders. Nevertheless, no studies have yet examined the bioavailability of PE–MPs. Similarly, the dose-response relationship between PE and MP exposure and toxicological outcomes in humans remains unclear. As a result, it is currently not possible to establish safety thresholds for PE–MP contamination in food products. This review offers a novel polymer-specific approach to MPs research and outlines specific recommendations for future studies.

## 1. Introduction

Plastic production reached 413.8 Mt in 2023. The most produced polymer was polyethylene (PE), accounting for 26.2% (108.42 Mt) [1]. In Europe, during the same period, 54.0 Mt of plastics were produced, with PE having the largest share at 21.4% [1]. It has been widely used for packaging consumer products across the food, pharmaceutical, chemical, and agricultural industries [2]. Given the increasing uncertainty surrounding polymer degradation and its impact on ecosystems and human health, it is crucial to focus on the most widely used polymer globally.

Extensive use of plastic materials has introduced an emerging contaminant into environment—microplastics (MPs). These synthetic polymer particles, with sizes ranging from 0.1 μm to 5.0 mm, are heterogeneous pollutants [3]. Their impact on the ecosystem is heavily influenced by the type of polymer, color, shape, size, degree of degradation, and route of exposure [4,5,6,7]. However, a detailed risk assessment, such as defining proper safety thresholds, has not yet been feasible for any polymer. To understand the severity of the risks associated with MPs and develop appropriate regulations, three key questions should be addressed, based on the available literature [3]:

1. Degradation of packaging—how are MPs generated; and how can their formation be prevented?
2. The role of MPs as carriers of contaminants—what is the extent of adsorption and desorption; and how can these processes affect human exposure?
3. Toxicity and bioavailability—what are the external and internal effects of MP exposure; and how can they be measured?

Microplastics of different polymers are often (though not always) found together in analyzed samples, with significantly varying ratios [8,9,10]. However, the properties of individual polymers and packaging types may lead to different effects in similar scenarios. For example, Tong et al. (2022) reported that PE pellets generated more MPs through mechanical degradation than via photooxidation, whereas PE bags exhibited the opposite effect, attributed to differences in thickness [11]. Dimassi et al. (2023) observed significantly greater degradation of polypropylene (PP) under marine conditions compared to poly(ethylene terephthalate) (PET) [12]. Wang et al. (2022) showed that PE–MPs had a higher adsorption capacity for tetracycline than poly(vinyl chloride) (PCV) MPs [13]. Similarly, Liu et al. (2022) reported that 10 μm PE particles had significantly greater adsorption capacity for the pesticide under study compared to 10 μm polystyrene particles [14]. Similarly, Jahan et al. (2024) observed that Nila tilapia were more sensitive to PET than PCV or PP following oral exposure, as evidenced by more severe physical and behavioral changes [15]. Therefore, to fully understand the risks associated with MPs and define appropriate safety thresholds, a more specific, polymer-based approach is necessary. This approach is gradually gaining popularity, as evidenced by recent reviews focused on specific polymers, such as PET [16].

The European Food Safety Authority (EFSA) recognizes PE–MPs as one of the most important and commonly encountered types of MPs [3]. Such particles are also considered to pose significant health concerns [17]. However, to the best of our knowledge, no comprehensive reviews on PE and PE–MPs that address these topics are currently available. The goal of this study was to review current literature data on PE degradation, the formation of PE–MPs, their adsorption and desorption properties, and their possible adverse effects. Particular attention was paid to the degradation of food packaging and implications for human health. This study aims to provide a comprehensive summary of current knowledge on PE as a source of MPs, identifying existing gaps and proposing novel research directions and approaches.

## 2. Materials and Methods

The literature review was based on scientific articles from ScienceDirect and PubMed databases. The search terms included combinations of “polyethylene” with “absorption”, “adsorption”, “bioaccessibility”, “bioavailability”, “degradation”, “equilibrium”, “microplastic”, and “packaging”. These terms were searched in titles, abstracts, and keywords. The analysis focused on peer-reviewed papers written in English and published after 2020 to ensure that the most up-to-date information was included. Selected articles published before 2020 were included due to their significance in PE and PE–MPs research. Approximately 1661 articles were identified, and 198 were ultimately selected for inclusion in this review. The primary selection criteria focused on studies examining PE as a source of microparticles. Particular attention was given to research addressing detection in various matrices and products, mechanisms of formation and degradation, roles as vectors for contaminants (including adsorption behavior, capacity, and influencing variables), as well as health-related outcomes such as bioaccessibility, distribution, and toxicity. Special attention was paid to publications on the topic of food contamination. Articles that did not meet those criteria were excluded.

## 3. Degradation of Polyethylene

Polyethylene, like other polyolefins, is generally considered an inert material. Due to the absence of additional polymerizable groups and its low polarity, PE exhibits very limited reactivity [18]. This characteristic contributes to PEs reputation as one of the safest synthetic polymers for food contact and biomedical applications. However, recent research shows that PE can degrade in the environment as a result of everyday human activities. This process may result in the release of hazardous additives, the formation of toxic organic compounds harmful to both humans and the environment, and the generation of PE-derived microplastics (PE-MPs) [19,20].

### 3.1. General Factors Influencing Degradation

**Figure 1:**
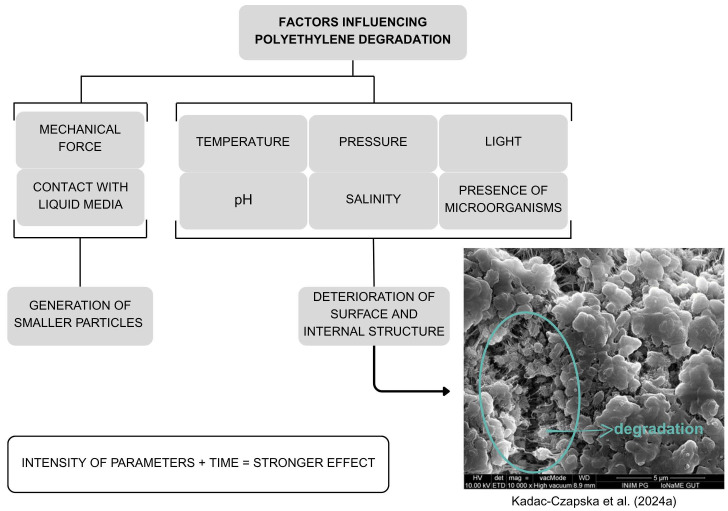
Effects of various parameters on polyethylene degradation. Developed by the authors based on [11,23,24,25,26,28,29,30].

The primary degradation pathway of PE is oxidation [21,22,23,24,25,26,27]. As shown in Figure 1, most degradation-accelerating factors lead to further material deterioration as the intensity of the influencing parameters increases. In general, the influencing factors can be categorized based on whether they involve physical contact or chemical interaction. Chemical degradation may result in the breakdown of polymer chains or the formation of new functional groups. However, the likelihood or extent of PE–MP generation through chemical degradation remains unclear [11,23,24,25,26,28,29,30].

The intensity of oxidation is significantly catalyzed by free radicals, such as ▪OH and O_2_^–^ [31,32]. These radicals may also promote the formation of smaller PE particles (0.2–0.5 μm) [32]. Additionally, advanced oxidation processes (heat–K_2_S_2_O_8_, ultraviolet–H_2_O_2_, ultraviolet-peracetic acid), commonly used in wastewater treatment systems, have been shown to increase surface area and pore volume while reducing the particle size of PE microparticles [33]. Similarly, in a study on the isolation of PE–MPs (34–50 μm in size) from sucrose-containing foods, Kadac-Czapska et al. (2024a) observed surface degradation of PE after exposure to HNO_3_ at concentrations of 1.9% or higher (5 min at 70 °C) [30]. Conversely, treatment with HCl solutions at concentrations up to 5.0% did not cause visible degradation, as confirmed by scanning electron microscopy and µ–Raman spectroscopy [30].

The oxidation of PE leads to the formation of new functional groups within its polymer chain, as well as the generation of various organic compounds and the release of additives. Fourier Transform Infrared Spectroscopy analysis shows new absorbance peaks for C–O stretch, C=O groups, OH groups, and CHO groups [34,35,36]. Organic compounds detected after the degradation of PE include aliphatic and oxygenated hydrocarbons, alcohols, aldehydes, ketones, esters, carboxylic acids, phenols, and lactones [20,31,37]. The released additives include diethyl phosphate, dimethyl phthalate, other phthalates, bisphenols, and siloxanes. These compounds may pose risks to both the environment and humans, raising safety concerns [20,25,31,37,38]. For instance, they may accumulate in soil and subsequently move up the food chain [5,39].

The stability of PE can depend on the ratio of virgin to aged or recycled PE used in the final product [40,41]. Ballestar de Las Heras et al. (2024) recommend limiting recycled PE in film applications (e.g., bags) to no more than four recycling cycles to preserve the desired properties of the final product [41].

### 3.2. Degradation Through Contact with Packaged Contents

Polyethylene is one of the most important packaging materials, used in various areas, including the food and pharmaceutical industries [42]. Understanding the potential interactions between PE packaging and its contents is essential to ensure product quality and consumer safety.

For example, acidic solutions, such as hot sauce (pH 2.7–3.1), have been found to degrade multilayer packaging [24,26]. In an accelerated 12-week immersion test conducted at 50 °C, three solutions were analyzed: Tabasco hot sauce (aged red pepper, distilled vinegar, acetic acid, and salt), 5 vol% acetic acid, and 12 wt% sodium chloride [26]. The study confirmed that both hot sauce and acetic acid degraded the packaging by converting aluminum into soluble salt, oxidizing the PE layers, and causing delamination. Tabasco sauce had a greater degrading effect than acetic acid, while the applied concentration of sodium chloride did not affect the packaging [26]. A similar degradation process was observed during a 12 months storage test using liquid and powdered hot sauce, as well as water, at 27 °C and 38 °C [24]. Liquid hot sauce induced more pronounced oxidation than the powdered form, which is a relevant observation in the context of food storage using PE and multilayer packaging [24]. In a study on low-density PE protective films used in liquid media containers, a low pH value was also identified as a degrading factor. For high-pH dish soap and disinfectant, the tensile strength at break in the transverse direction increased by 33.02% and 14.06%, respectively, possibly due to crosslinking of polymer chains. In contrast, low-pH Coca-Cola reduced this parameter by 36.90%, likely due to bond cleavage [2]. The potentially beneficial effect of high pH on packaging stability is noteworthy and warrants further investigation. Nevertheless, most foods (with few exceptions, such as egg whites) are naturally acidic. Moreover, acidifying agents are often added to food products for preservation purposes [43]. Therefore, assessing the impact of low pH on packaging degradation and MP generation should be prioritized.

Contact with synthetic polymer materials, including PE, has also been shown to promote hydroperoxide formation in tested oils. The amount of detected hydroperoxide was related to the hydrophilicity of polymers, with higher values observed for less hydrophilic surfaces. In addition, PE accelerated hydroperoxide decomposition, producing more secondary volatile products [44].

### 3.3. Degradation Through Sterilization

Research on the interaction between PE packaging and its contents in the medical and pharmaceutical industries remains limited. Girard–Perier et al. (2020) investigated the effects of gamma irradiation sterilization on single-use multilayer bag seals, one of which consisted of a PE/ethyl vinyl alcohol/PE film [45]. Tensile behavior remained unchanged, and no variation in seal resistance was observed for irradiation doses up to 115 kGy. However, alterations were detected at a dose of 270 kGy [45]. The standard dose used in industrial sterilization processes (30–55 kGy) does not appear to alter the tensile or chemical properties of PE packaging. However, molecular weight changes indicate a tendency towards cross-linking [46]. The studies cited did not assess the formation of MPs [45,46]. Analyzing the release of PE-derived MPs under various sterilization conditions would improve understanding of the mechanisms behind their generation and the potential contamination of products, especially since sterilization is often performed on pre-packaged goods.

### 3.4. Release of PE–MPs Through Packaging

The formation of MPs follows the degradation mechanisms of their respective polymers, although some factors may vary depending on the properties of the material. For instance, Tong et al. (2022) found that mechanical forces played a more significant role in generating MPs from primary pellets compared to photooxidation, whereas photooxidation was the dominant factor in the case of PE bags [11].

The degradation of PE packaging and subsequent release of PE–MPs may be accelerated by the temperature of its contents. Hee et al. (2022) compared the effects of a 1 hour hot water treatment at 95 °C and a 1 hour cold water treatment with ice on PE takeaway bags [28]. Compared to cold treatment, hot water exposure resulted in over a twofold increase in MPs release from PE packaging [28]. However, Samal et al. (2023) reported mixed results for PE bags and PE-coated paper cups, with the former releasing more MPs when exposed to water at 4 °C [47]. In both cases, Nile Red staining was used for the quantification of MPs [28,47]. However, Nile Red-based identification and quantification methods carry a risk of false positives due to the presence of naturally occurring particles [48]. Additional testing under room temperature conditions would help determine whether MPs release is driven by temperature exposure or solely by the food simulant.

In another study, plastic PE coffee bags were found to release small-sized polymer particles even after being steeped for only 5 min at 95 °C [49]. However, in a study by Mei et al. (2022), soaking conditions had no significant effect on particle release in tea filter bags [50]. Similar particle release was also observed during simulated use of PE breastmilk storage bags [51].

### 3.5. Occurrence and Detection of PE–MPs

The presence of MPs has been reported worldwide across various types of samples. Special attention should be given to PE, as it is frequently identified as the most abundant polymer in tested food samples. For instance, Bai et al. (2022) identified PE as the predominant MPs polymer type in take-out food [52]. According to their research, the amount of MPs varied significantly depending on storage conditions, food types, and cooking methods [52]. Polyethylene MPs were also detected in 100% of the samples analyzed by Visentin et al. (2024) in beef hamburgers [53] and by Dessì et al. (2021) in packaged rice [54]. Table 1 summarizes the most relevant findings regarding the occurrence and detection of PE–MPs.

## 4. Polyethylene as a Carrier of Contaminants and Its Interactions

### 4.1. Adsorption on Polyethylene Microplastics

Adsorption refers to the adhesion of atoms, ions, or molecules from a gas, liquid, or dissolved solid onto a surface, forming a film of the adsorbate on the adsorbent material [63]. In contrast, desorption refers to the release of the adsorbate from the adsorbent [64].

Polyethylene MPs have been shown to adsorb a wide range of substances, including organic and inorganic compounds [65,66,67,68] as well as microorganisms [69,70]. Table 2 presents detailed results and parameters of various studies on the adsorption of organic and inorganic substances on PE–MPs with a focus on maximum adsorption capacity and conditions. In general, available data suggest higher sorption capacities for organic compounds than inorganic elements. Among organic compounds, the highest sorption capacities were reported for lambda-cyhalothrin, norfloxacin, and tetracycline. Among inorganic elements, the highest sorption capacities were reported for lead (Pb^2+^) and cadmium (Cd^2+^).

Due to their structure, PE–MPs can offer unique microbial attachment sites. The presence of such particles in the environment may lead to the formation of specific bacterial communities enriched in antibiotic resistance genes [101]. In one study, PE–MPs exhibited stronger adsorption of *Enterococcus faecalis* than stainless steel, likely due to their higher hydrophobicity. This interaction increased the bacteria’s resistance to ceftriaxone [69]. Adsorption of *Escherichia coli* by PE–MPs was also shown to decrease the disinfection efficiency of sodium hypochlorite [70]. Moreover, microbial activity may alter the structure of PE–MPs, thereby enhancing their capacity to adsorb environmental contaminants [78].

#### 4.1.1. Mechanisms and Factors Influencing Adsorption on Polyethylene

**Figure 2:**
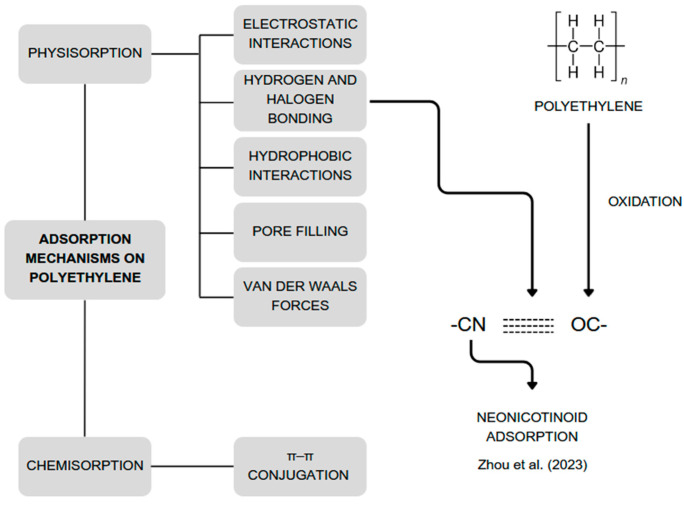
Mechanisms involved in the adsorption of various substances on polyethylene. Developed by the authors based on [13,14,65,66,103,104,105].

Studies show that adsorption on PE particles is endothermic and spontaneous [66,87,102]. Various adsorption mechanisms on PE are illustrated in Figure 2. These mechanisms can be classified as either physical or chemical in nature. Physical mechanisms do not result in a permanent change in the structure of adsorbent or adsorbate. However, structural changes caused by external conditions can affect the adsorption mechanisms. For instance, the –C=O groups generated through oxidation of PE polymer chains can form hydrogen bonds with the –CN group of neonicotinoids, enhancing their adsorption [13,14,65,66,103,104,105].

The process can be inhibited by ultraviolet irradiation, as observed for organotins [106] and tetracycline [107]. Ionic strength inhibited the adsorption of norfloxacin [103], ciprofloxacin [72], flusilazole, and epoxiconazole [14], but promoted adsorption of tetrabromobisphenol A [83] and had no effect on adsorption of 9-nitroanthracene on PE–MPs [92]. A potential effect promoting adsorption can occur for heavy metals due to the influence of tropical temperature [68]. However, for sulfamethoxazole, adsorption capacity tends to rise with temperature from 5 °C to 20 °C and decrease from 20 °C to 30 °C [75]. Depending on the contaminant, the influence on adsorption can vary due to pH level [66,68,92,108], pKa [108], and salinity [74,75,77,93].

#### 4.1.2. Influence of Other Substances on the Adsorption Process

The coexistence of other substances can significantly modulate the adsorption behavior of PE–MPs. Prothioconazole inhibited adsorption of chromium (Cr), arsenic (As), lead (Pb), and barium, but promoted adsorption of copper (Cu) and had no significant effect on tin [109]. The adsorption of tetracycline was promoted in the presence of Cr^3+^, cadmium (Cd^2+^), Pb^2+^, and zinc (Zn^2+^), but was weakened in the presence of Cu^2+^ [110]. Cephalexin increased, decreased, or had no effect on Cd adsorption, depending on the concentration of the antibiotic [93], while sodium dodecyl benzene sulfonate had different effects on Cr^6+^ adsorption depending on pH [96]. Dissolved organic matter, which includes humic substances [83], had an inhibiting effect on the adsorption of norfloxacin [103]. However, in another study, adsorption of norfloxacin decreased with increasing concentrations of humic acid up to 10 mg/L, after which adsorption began to increase with further increases in the acid concentration [74]. Oxalic acid inhibited the adsorption of bensulfuron-methyl [66]. Fulvic acid inhibited the adsorption of carbamazepine, bisphenol A, estrone, triclocarban, 4–tert–Octylphenol [111], as well as Pb [100]. However, it could promote the adsorption of Pb on PE–MPs in sediment [112]. Oxalic acid and humic acid decreased adsorption of flusilazole [14]. Humic acid decreased the adsorption of tetrabromobisphenol A [83], ciprofloxacin [72], atrazine, and imidacloprid [87], but increased the adsorption of radionuclide U–232 [113] and Pb [100]. The mixed results for adsorption of various combinations of substances require further study.

#### 4.1.3. Influence of Erosion and Weathering on the Adsorption Process

Environmental erosion of PE–MPs, or its absence, can lead to opposite effects on the adsorption of organic and inorganic pollutants. Junck et al. (2024) found approximately 245% greater sorption of terbuthylazine by virgin PE–MPs (<250 μm) compared to aged particles [67]. However, aged PE–MPs adsorbed approximately 300% more Pb^2+^ than virgin microparticles. This was attributed to the high affinity of terbuthylazine for non-polar surfaces and to the increased polarity of the surface of PE–MPs due to degradation [67].

Ho et al. (2023) observed increased adsorption of tetracycline on 48 μm PE–MPs treated with strong oxidizing agents (KMnO_4_, NaOCl) and decreased adsorption in samples treated with weaker oxidizers (H_2_O_2_, Na_2_S_2_O_8_), compared to untreated PE [79]. This was attributed to the melting and smoothing of the particles by the oxidizing agents with lower oxidation potentials [79].

The adsorption of organic compounds can be significantly influenced by their polarity. The hydrophilicity of PE–MPs increases with progressive weathering, leading to greater adsorption of hydrophilic substances [114].

Additionally, the sorption properties of PE–MPs can influence the interaction of pollutants with other adsorbents. In hyporheic zone sediments, the presence of PE–MPs extended the adsorption equilibrium time of oxytetracycline from 24 to 36 h [115].

#### 4.1.4. Adsorption Equilibrium

According to the available data, the adsorption equilibrium for PE–MPs is most often reached after 48 h of exposure. This applies to both organic substances and inorganic elements, with the adsorption capacity varying depending on the type of polymer [103,116]. Such results have been reported for the adsorption of 9-nitroanthracene [92], chlorobenzenes and trifluralin [86], norfloxacin [103], tetracycline [13], Cr(III) and Cr(VI) [116], and mercury(II) [117] on various MPs, including PE–MPs. However, different adsorption equilibrium times have also been reported, such as 3 h for ciprofloxacin (1 h with the addition of humic acid) [72], 6 h for tetrabromobisphenol A [83], 8 h for imidacloprid [87], 24 h for atrazine [87] and tetracycline [79,102], 30 h for tetracycline [71], 72 h for chlorophenylacetonitriles [118], 96 h for triclosan [81], 120 h for four As species [119], 21 days for amoxicillin [120], and 28 days for phenol [120]. Differences in results could be attributed to varying experimental conditions, tested compounds, polymer sizes, and reagent concentrations. For instance, Zhang et al. (2024) observed a sorption equilibrium time of 6 h for bensulfuron-methyl at 500 mg/L of PE–MPs (0.5 μm) and 12 h at 2000 mg/L of PE–MPs (0.5 μm) [66]. Observed discrepancies could also suggest that the time needed to achieve full adsorption capacity depends on the substance that is adsorbed. In any case, those findings are mostly important in the environmental context or as a background for future design of studies on adsorption on PE–MPs. In the context of foods and pharmaceuticals, more attention should be given to the desorption of adsorbed substances. This includes their release either into the contaminated products (e.g., due to external contamination of products with PE–MPs from the environment) or into the human body after ingestion.

#### 4.1.5. Desorption and Influential Factors

Adsorbed contaminants may be released into the environment or after ingestion of MPs, highlighting their potential role as pollutant carriers and associated health risks [121,122]. This property is particularly relevant in the context of human health and oral exposure to MPs. The pH and the presence of gastric enzymes can affect the sorption behavior of PE–MPs, often promoting desorption [104,107,123,124]. For instance, McDougall et al. (2022) reported that PE–MPs preferentially adsorb hydrophobic pharmaceuticals in their cationic form, while anionic compounds are repelled due to the negative surface charge of PE–MPs, resulting in lower adsorption [104]. However, exposure to gastric fluids at 37 °C and pH 2 significantly reduced the surface charge of MPs, promoting the desorption of cationic pharmaceuticals. Fluoxetine, for example, showed up to 50% desorption under these conditions [104]. This study demonstrates a mechanism by which PE–MPs adsorb contaminants and subsequently release them upon human contact, acting as potential vectors of chemical exposure. Additionally, Fan et al. (2022) reported that the desorption rate of tetracycline from PE–MPs into simulated intestinal fluid increased to 72.10% compared to 25.16% in water [124]. The influence of specific factors largely depends on the chemical nature of the desorbed substance. Liu et al. (2020) observed physical entrapment of nonionic, nonpolar pyrene within the porous domains of MPs, suggesting that polymer rigidity plays a key role in desorption and bioaccessibility [125]. In contrast, the polarity of MPs and polar interactions were key determinants of 4-nonylphenol desorption [125]. Furthermore, both polarity and rigidity were influenced by the aging processes of MPs [125].

However, greater adsorption of a compound does not necessarily translate into higher absorption in the human body. Hu et al. (2022) found that phenanthrene derivatives with lower hydrophobicity and higher water solubility exhibited greater bioaccessibility in body fluids than their more hydrophobic counterparts, which were adsorbed in larger quantities [105]. This suggests that certain organic pollutants may pose higher health risks than others, even if their adsorption onto MPs is lower [105].

#### 4.1.6. Kinetic and Isotherm Models

Numerous studies have shown that adsorption on PE–MPs follows a pseudo-second-order kinetic model and aligns with the Freundlich isotherm [14,66,71,126,127,128,129,130]. This indicates that adsorption on PE–MPs is primarily driven by adsorbent-adsorbate interactions. The adsorption rate increases with the concentration of the adsorbate but levels off at higher concentrations, consistent with multilayer adsorption on heterogeneous surfaces [131,132,133].

However, some studies report data that fit alternative models, including the Langmuir (monolayer sorption) [119], Redlich–Petersen model [134], parabolic diffusion model, Elovich, Hill, and Dubinin–Radushkevich models [72]. These discrepancies may be explained by the presence of plastic additives [119] and humic substances [72]. The Redlich–Petersen model, which combines characteristics of both Freundlich and Langmuir isotherms, suggests that monolayer and multilayer adsorption can occur simultaneously [134]. This may explain why some studies apply two different models to describe the same substance [102,120,130]. Due to the complex nature of adsorption processes, the influence of secondary factors is often not immediately evident. Interestingly, the Langmuir isotherm and pseudo-first-order kinetics were reported for the adsorption of antibiotics on PE cut into macro-scale pieces [108]. Naturally, such plastic fragments differ in surface structure and physicochemical properties from 10 μm to 100 μm particles [14,134], which may justify the use of distinct adsorption models.

### 4.2. Interactions Between Polyethylene Microplastics and Other Substances

The co-occurrence of various substances in the environment can influence the adsorption process onto PE–MPs. Budhiraja et al. (2022) observed significantly higher triclosan adsorption onto virgin and oxidatively degradable PE films in a methylparaben-triclosan mixture compared to triclosan alone. Conversely, methylparaben adsorption was not enhanced in the mixture, with only the degree of weathering (i.e., degradation level) influencing the process [114]. The authors proposed that methylparaben, due to its affinity for weathered surfaces, adsorbs first and alters the MPs surface structure, thereby enhancing triclosan adsorption [114].

Polyethylene MPs can exert stronger adverse effects when combined with other organic or inorganic substances. However, it remains unclear whether these effects are synergistic or merely cumulative. The combination of PE–MPs and sulfanilamide more strongly reduced total chlorophyll content in *Vallisneria natans* than either pollutant alone, particularly in the 0.5% PE–MPs + 1 mg/L sulfanilamide treatment [135]. Additionally, 1% PE–MPs increased Cd bioavailability in soil (30-day incubation) [136], enhanced Cr uptake by cucumber plants [137], and promoted as mobility and bioavailability in river sediments (65-day incubation) [138]. In *Microcystis aeruginosa*, combined exposure to 0.2 mg/L Cd and 100 mg/L PE–MPs (6.5 and 13 µm) caused increased oxidative stress and significantly inhibited chlorophyll content, which dropped to zero by day 5 [139]. In *Danio rerio*, neurotoxicity and oxidative stress were greater for the 9-nitroanthracene-PE–MPs complex than for 9-nitroanthracene. Moreover, PE–MPs acted as carriers, facilitating pollutant transport into zebrafish; however, bioaccumulation was reduced due to surface adsorption [140].

Another interaction mechanism between PE–MPs and contaminants is stabilization via surface adsorption. For example, PE–MPs stabilized triclosan and inhibited its biodegradation, leading to greater accumulation in mussels [141].

However, interactions between PE–MPs and other substances do not always exacerbate toxic effects. In fact, PE–MPs reduced the toxicity of niclosamide, a molluscicide used to control snail populations in aquatic environments [142]. Additionally, the adsorption of sulfamethoxazole by PE–MPs appeared to reduce its toxicity to marine algae [143].

## 5. Toxicity of Polyethylene

### 5.1. Bioaccessibility

**Figure 3:**
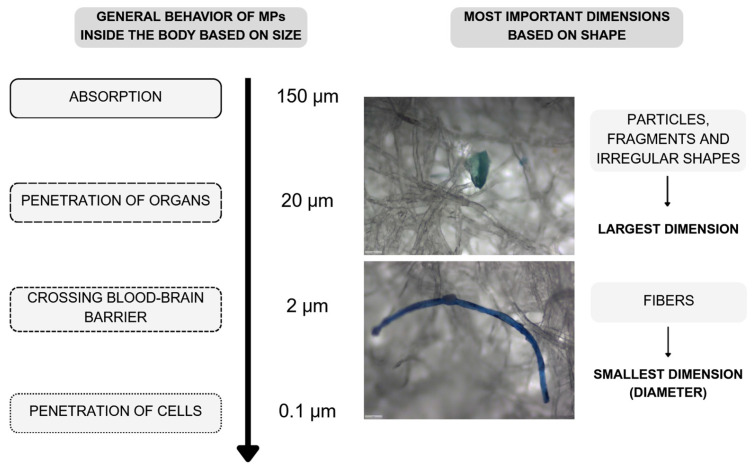
General behavior of MPs inside the body based on their size. Developed by the authors based on [3,4,57,144,145] and own unpublished data.

Currently, a substantial knowledge gap remains regarding the bioaccessibility of MPs in humans, particularly PE–MPs. The limited available data suggest that the bioaccessibility of MPs primarily depends on particle size, as illustrated in Figure 3. Smaller particles are more likely to penetrate biological membranes and accumulate in the body, posing potential health risks [3,4,144,145]. To date, no studies have specifically addressed this issue in the context of PE–MPs.

Data on the absorption of MPs from the gastrointestinal tract remain scarce, and no studies have specifically addressed the absorption of PE–MPs. The only study related to the oral consumption of PE–MPs by humans involved a single dose of 15 g of 1–2 mm sized particles added to rice pudding. This resulted in accelerated small bowel transit, comparable to that observed with coarse bran. No absorption of PE–MPs was observed [3,146]. Therefore, the following paragraph includes data on other polymers to inform future research on PE–MPs.

In 2016, EFSA released a statement based on rodent and human ex vivo models concerning the presence of MPs in food and seafood. The report indicated a possible gastrointestinal absorption rate of 0.04–0.3% for particles smaller than 150 μm and intestinal uptake for particles in the 2–3 μm range [3,147].

Particles larger than 150 μm are unlikely to be absorbed through the gastrointestinal tract and may instead induce local immune responses and inflammation [3,4]. Nonetheless, even among the few available studies, the data varied considerably. In general, predicting MPs uptake is challenging, as not only particle size but also composition, surface charge, and hydrophilicity significantly influence the process [3]. Therefore, future research should include other particle types commonly found in food, with particular attention to their shape, size, and degree of degradation. Such research would also benefit from the development of appropriate in vitro models that replicate the different phases of absorption in the human body, as animal models are difficult to scale and translate to human physiology. Ethical considerations related to animal and human studies should also not be overlooked. An example of an in vitro digestion system is the comprehensive digestive model developed by Yu and Yang (2019) [148]. This model simulates digestion in four distinct phases: oral, gastric, small intestinal, and large intestinal. Each phase contains synthetic digestive fluids that replicate natural saliva, bile, and gastric, duodenal, and large intestinal secretions [148]. Such models could be further refined to analyze the bioaccessibility of MPs.

### 5.2. The Fate of Microplastics After Ingestion

To date, no reports have described the extent of PE–MPs absorption via dermal contact or inhalation. However, according to Prata, low-density polymers such as PE have a greater potential to reach the lower respiratory tract in humans [149]. Additionally, some MPs have been confirmed to penetrate pulmonary barriers and even accumulate in cardiac tissue [150]. A deeper understanding of this issue could be gained by applying methodologies used to analyze the permeation of other MPs or pharmaceuticals, for example, through three-dimensional skin [151] and lung models [152].

It is important to note that the shape of MPs influences their behavior within the body. For example, although fiber length affects their distribution, diameter is a more critical factor. According to Donaldson et al. (1993), fibers with diameters of 3 μm or less can deposit in the alveolar region of the lungs [153].

Most MPs that enter the body remain chemically and physically unchanged during gastrointestinal transit. Stock et al. (2020), using an in vitro model, tested the effects of gastrointestinal digestion on various particles, including PE with an average size of 90.1 μm [154]. None of the particles underwent changes in size or shape due to digestive fluids, nor were they decomposed. The only observed effect was the formation of a protein corona on the particle surfaces. This subsequent increase in particle size could potentially hinder absorption [154,155]. Additional evidence supporting the chemical stability of MPs during digestion includes their identification (including PE–MPs) in human stool [156,157] and urine samples [158]. Nevertheless, some smaller MPs may become trapped in the body and accumulate over a lifetime. Mohamed Nor et al. (2021) estimated the potential accumulation of 1–10 μm particles in body tissues to be 8.32 × 10^3^ particles per capita for children up to age 18 and 5.01 × 10^4^ particles per capita for adults up to age 70 [159]. The significance of this issue is further underscored by the discovery of MPs in human blood, placenta, and even meconium [160,161,162]. It should be noted that the estimates by Mohamed Nor et al. (2021) include all types of MPs [159]. In the future, assuming significant differences in toxicity between polymer types or categories (e.g., non-biodegradable vs. biodegradable or amorphous vs. crystalline), more detailed assessments of exposure to each MP type should be considered.

### 5.3. Health Implications for Humans

The growing concern over the dangers posed by small polymer particles is unsurprising, as multiple studies have reported potential negative health effects in humans, even from PE–MPs alone. Polyethylene particles were detected in 20 % of thrombus samples from patients with myocardial infarction and in 25% of those with ischemic stroke in China. No significant correlation was found between thrombus size and MP concentration. However, among ischemic stroke patients, those with higher MP concentrations had significantly elevated NIH Stroke Scale scores compared to those with lower concentrations [163]. A separate study of Italian patients reported a higher risk of a composite outcome—myocardial infarction; stroke, or all-cause mortality—after 34 months of follow-up in individuals with polymer particles (predominantly PE) detected in carotid artery plaques; compared to those without such findings [164]. In a three-phase simulated digestion model, DeLoid et al. (2022) reported a 33% increase in fat digestion and 147% and 145% increases in fat absorption at 1 and 2 h, respectively, following exposure to 400 μg/mL of laboratory-generated PE particles (~0.1 μm) produced via controlled incineration [165].

In a recent study, Nihart et al. (2025) found substantial amounts of MPs in human tissues, including samples from the liver, kidney, and brain [166]. Polyethylene particles were the most prevalent, with their occurrence ranging from 40.3 to 74.5% [166]. Another finding was that plastic concentrations were higher in samples collected from individuals who died in 2024 compared to those who died in 2016. Additionally, brain samples from deceased patients diagnosed with dementia contained nearly an order of magnitude more plastic particles than those from patients without dementia [166]. However, the small sample size used in the study limits the generalizability of its findings. Caution is warranted in interpreting these results due to limitations associated with pyrolysis-gas chromatography/mass spectrometry (Py–GC/MS) analyses. For example, fats yield the same pyrolysis products as PE, meaning that a high fat content in analyzed samples could lead to an overestimation of PE–MPs content. Furthermore, this method does not allow determination of MPs size or shape, which complicates health risk assessment [167].

Moreover, a wide range of particle sizes and exposure durations to PE–MPs has been shown to reduce both sperm quality and quantity [158,168]. Microplastics may also be linked to bowel diseases. In a study by Yan et al. (2022), patients with irritable bowel disease exhibited significantly higher overall concentrations of MPs in their stool compared to healthy individuals (41.8 MPs/g dry weight vs. 28.0 MPs/g dry weight) [157]. However, the concentrations of specific polymers varied between patient groups, with a higher percentage of PE–MPs observed in healthy individuals [157].

In this context, it remains unclear whether different polymer types exert significantly different health effects. Notably, the study by Yan et al. (2022) included questionnaires on patients’ drinking and dietary habits, as well as their living and working conditions [157]. Patients exposed to bottled water, takeaway food, and dust had substantially higher MPs concentrations [157]. Including such lifestyle surveys in future studies is strongly recommended to assess the potential influence of environmental and behavioral factors. As Marfella et al. (2024) rightly noted, a high presence of MPs may not necessarily be the direct (or sole) cause of a patient’s condition but rather an indicator of environmental exposure, lifestyle, overall health status, and co-exposure to other hazards such as air pollution [164].

Although knowledge on this topic remains limited, the dermal exposure pathway should also be considered for PE–MPs. At the very least, flame-retardant additives used in their production may be absorbed through the skin [169,170].

Emphasis should be placed on investigating the potential role of PE–MPs in common lung, heart, brain, and gastrointestinal diseases, as well as in obesity and infertility, as suggested by current preliminary findings.

Toxic effects of PE particles have been observed in various in vivo and in vitro models, including animals, plants, and cell cultures; however, their relevance to human health remains to be determined. One of the earliest studies on PE–MPs toxicity involved the earthworm *Lumbricus terrestris*, which was exposed to particles smaller than 150 μm at varying concentrations. Oral exposure resulted in significantly increased mortality and reduced growth rate, both by up to 60%, after 60 days of experimentation [171]. In honeybees, exposure to 100 μm PE–MPs altered gut microbiota and increased mortality, most likely due to damage to epithelial cells and the peritrophic membrane layer. However, this effect was not observed for particles 1–10 μm in size, which only caused a reduction in intestinal wall thickness without significant effects on survival or body weight [172].

Furthermore, altered gut morphology and inflammation were reported in mice following oral exposure to 36 μm and 116 μm microbeads, with stronger effects observed in co-exposure treatment involving both sizes [173]. Microparticles measuring 10–45 μm in diameter were transferred to neonates following intratracheal exposure of dams. In the high-dose group (60 µg/mouse/day), particles were detected only in the kidneys [174]. Additional detailed information on the effects of exposure to PE particles in animal, plant, and cell models is provided in Table 3. Notably, the studies mentioned had relatively short experimental durations, which may limit the extrapolation of their findings to long-term exposure scenarios.

The primary mechanism of toxicity by which PE–MPs can damage cells is the generation of reactive oxygen species [190]. However, the effects are not always dose-dependent and vary depending on the cell type. Schirinzi et al. (2017) reported negative effects to human cerebral cells (T98G) only at concentrations of 0.05 mg/L and 0.1 mg/L, but not at 1 mg/L or 10 mg/L [190]. Additionally, PE–MPs affected only T98G, but not human epithelial cells (HeLa) [190].

Another important concern is the potential for MPs to interfere with endocrine function, leading to hormonal imbalances or disruptions. In a recent study, Ye et al. (2025) analyzed the endocrine-disrupting effects and cellular toxicity of PE–MPs occurring alone or accompanied by bisphenol A [191]. Researchers found a significant impact on the viability of MLTC–1 cells when exposed to 20 μm sized PE particles, but only at concentrations above 100 μg/mL. However, co-exposure to PE–MPs and bisphenol A resulted in a more substantial decrease in cell viability than bisphenol A alone [191]. In the same study, even single exposures of zebrafish to PE–MPs resulted in changes indicative of endocrine disruption, such as alterations in gene expression related to hypothalamus-pituitary-gonad or an increased gonadosomatic index [191]. This study provides new insights into the potential effects of combined exposure to PE–MPs and known endocrine-disrupting chemicals, such as bisphenol A. The use of additional in vivo models to validate these findings would be beneficial, along with expanding the research to include other polymers and contaminants [191].

Further research is needed to fully understand the dose and duration of exposure to specific types of MPs that induce harmful effects. Of particular importance is the thorough examination of chronic toxicity, as studies of this kind are considerably less common.

As pointed out by Chen et al. (2024), aged MPs are more representative of the actual environmental conditions than virgin MPs [7]. Therefore, their use is recommended in toxicological studies [7].

### 5.4. Environmental Contamination and Its Dangers

The negative effects on animals are not the only reason why the widespread presence of PE in the environment is concerning. Although European countries have demonstrated a steady increase in recycling and energy recovery, a significant proportion of produced plastic still ends up in landfills or incinerators, posing environmental risks. Moreover, the increasing share of global plastic production in China and other parts of Asia highlights a broader issue: Outsourcing manufacturing instead of phasing out hazardous processes and materials on a global scale. This is further exemplified by the modest decrease in plastic consumption in Europe—only 3.8% between 2018 and 2022 [42].

Polyethylene particles can contribute to the emission of greenhouse gases, such as CO_2_, CH_4_, and N_2_O, with their impact potentially exceeding that of other non-biodegradable plastics [192]. However, some studies suggest that this effect may be even more pronounced in the case of biodegradable synthetic polymers [193,194,195]. Additionally, the presence of PE particles can adversely affect soil properties and the microbiome, often increasing the abundance of bacteria and fungi capable of degrading these polymers [196,197,198].

## 6. Conclusions

A thorough risk assessment and analysis of the factors influencing the release of MPs, particularly PE, from packaging is necessary, given the frequent detection of such particles in food. This would help minimize human exposure to MPs. Temperature, pressure, pH, salinity, oxidation, time, light, mechanical force, and the presence of microorganisms all play vital roles in the degradation of PE. Nonetheless, the exact extent of their influence requires further study. More detailed research on the sorption properties of PE–MPs, focusing on interactions between specific MPs (size, shape, polymer type, amount/concentration) and pollutants under clinically relevant conditions, would be beneficial for improving our understanding of potential human health risks. Special attention should be given to the desorption of adsorbed contaminants following ingestion of PE–MPs. The significant lack of detailed knowledge regarding the fate of PE–MPs in the human body following dietary exposure is a matter of concern.

Future studies should not focus on MPs as a collective category but rather consider each combination of size, shape, and polymer type as a distinct contaminant with unique properties. Understanding the impact of each MPs category will facilitate more precise insight into their interactions. Given the reported potential effects of PE and other MPs on various lifestyle-related diseases, such knowledge would contribute to public health preservation efforts. This review provides a comprehensive overview of current knowledge regarding PE as a prevalent microparticle contaminant. It is recommended to analyze other synthetic polymers in a similar manner to better understand the risks associated with different MPs and their co-occurrence.

## Figures and Tables

**Table 1:** Occurrence and detection of PE–MPs in foods.

Source of MPs	Amount Detected—Average/Range (All MPs)	Percentage of PE–MPs [% of the Total]	Size Range [μm] (All MPs)	Most Abundant Shape (All MPs)	Reference
Beef hamburgers, Italy	unspecified 200–30,300 MPs/kg	17.4	30–3154	Fragments; 95.99%	[53]
Bottled water, UK	37 ± 11 MPs/L 12–62 MPs/L	20.0–40.0	11.36–98.90	Fragments; 72.0%	[55]
Eggs, Wuxi, Jiangsu Province, China	11.67 ± 3.98 MPs/egg unspecified	100.0	50–100	Pellets	[10]
Ice cubes, Mexico City, Mexico	79 ± 47 MPs/L 19–178 MPs/L	24.0	20–>500	Fibers; 87.0%	[56]
Infant formula, Poland	42 ± 27 MPs/100 g 7–130 MPs/100 g	27.7	6–4380	Fibers; 61.1%	[57]
Omega-3 oil supplements (raw and capsules), South Korea	1.2 ± 1.7 MPs/g–10.6 ± 8.9 MPs/g unspecified	0–5.0	5–>100	unspecified	[58]
Salt, Iran	1278 ± 553–1825 ± 1808 MPs/kg 700–5470 MPs/kg	20.0	>0.45	Fragments; 51.0–61.0%	[59]
Seaweed, Mexico City, Mexico	24.0 ± 9.4 MPs/g 4–64 MPs/g	unspecified	17–1647	Fibers; 61.0%	[60]
Soft drinks and non-alcoholic beverages, Kermanshah, Iran	21.90 ± 25.72 MPs/L 0–120 MPs/L	unspecified	1–1500	Fragments; 71.0%	[61]
Tap water, UK	40 ± 16 MPs/L 6–100 MPs/L	20.0–50.0	10.98–320.39	Fragments; 67.0%	[55]
Teabags—German brand	147.28 MPs/teabag 55.6–323.2 MPs/tea bag	53.9	20–5000	Fibers; 99.99%	[19]
Teabags—Persian brand	412.32 MPs/teabag 73.6–1446.8 MPs/tea bag	53.9	20–5000	Fibers; 99.99%	[19]
Vegetable edible oils, Italy and Spain	1140 ± 350 MPs/L 644–1795 MPs/L	50.3	20–>500	Fragments; 81.2%	[62]

**Table 2:** Adsorption of compounds and elements on PE–MPs.

Compound/Element	Compound/Element Concentration	PE–MPs Size	PE–MPs Concentration	Conditions	Sorption Capacity	Reference
**Adsorption of organic compounds**						
Antibiotics						
Chlortetracycline	10 mg/L	150–425 μm	10 g/L	25 °C; pH = 5; 30 h	0.06336 ± 0.00492 mg/g	[71]
Ciprofloxacin	25 mg/L	100 μm	2 g/L	25 °C; pH = 6.5–7.5; 5 h; 150 rpm shaking	2.1 mg/g	[72]
Levofloxacin	10 mg/L	125 μm	0.5 g/L	25 °C; pH = 6–7; 72 h; 150 rpm shaking; NaCl = 0.00 M	0.35 mg/g	[73]
Norfloxacin	15 mg/L	75 μm	0.5 g/L	15 °C; pH = 7; 24 h; 120 rpm shaking	13.26 mg/g (virgin PE–MPs)	[74]
Norfloxacin	15 mg/L	75 μm	0.5 g/L	15 °C; pH = 7; 24 h; 120 rpm shaking	10.36 mg/g (ultraviolet–aged PE–MPs)	[74]
Oxytetracycline	10 mg/L	150–425 μm	10 g/L	25 °C; pH = 5; 30 h	0.06440 ± 0.00238 μg/g	[71]
Sulfamethoxazole	25 mg/L	75–100 μm	20 g/L	20 °C; 24 h; 220 rpm shaking; salinity = 0.05%	0.106 ± 0.004 mg/g	[75]
Sulfamethoxazole (anion)	0.050 mg/L	45–48 µm	0.2 g/L	25 °C; pH = 7; 96 h; 150 rpm shaking	0.00783 mg/g	[76]
Sulfamonomethoxine	10 mg/L	unspecified	0.00813 g/L	25 °C; pH = 7; 3 h	0.0955 mg/g	[77]
Tetracycline	10 mg/L	150–500 μm	6.666 g/L	25 °C; pH = 7; 60 h; 110 rpm shaking	1.0953 mg/g (microbial–aged PE–MPs)	[78]
Tetracycline	10 mg/L	150–500 μm	6.666 g/L	25 °C; pH = 7; 60 h; 110 rpm shaking	0.1627 mg/g (H_2_O_2_-treated PE–MPs)	[78]
Tetracycline	10 mg/L	150–500 μm	6.666 g/L	25 °C; pH = 7; 60 h; 110 rpm shaking	0.1336 mg/g (untreated PE–MPs)	[78]
Tetracycline	10 mg/L	150–425 μm	10 g/L	25 °C; pH = 5; 30 h	0.05352 ± 0.00343 mg/g	[71]
Tetracycline	9.161 mg/L	48 μm	1 g/L	25 °C; pH = 7; 24 h; 200 rpm shaking; NaNO_3_ = 0.01 M	11.099 mg/g (KMnO_4_-treated PE–MPs)	[79]
Tetracycline	9.161 mg/L	48 μm	1 g/L	25 °C; pH = 7; 24 h; 200 rpm shaking; NaNO_3_ = 0.01 M	9.117 mg/g (NaOCl-treated PE–MPs)	[79]
Tetracycline	9.161 mg/L	48 μm	1 g/L	25 °C; pH = 7; 24 h; 200 rpm shaking; NaNO_3_ = 0.01 M	7.223 mg/g (untreated PE–MPs)	[79]
Tetracycline	9.161 mg/L	48 μm	1 g/L	25 °C; pH = 7; 24 h; 200 rpm shaking; NaNO_3_ = 0.01 M	4.052 mg/g (H_2_O_2_-treated PE–MPs)	[79]
Tetracycline	9.161 mg/L	48 μm	1 g/L	25 °C; pH = 7; 24 h; 200 rpm shaking; NaNO_3_ = 0.01 M	1.233 mg/g (Na_2_S_2_O_8_-treated PE–MPs)	[79]
Tetracycline	10 mg/L	40–48 μm	1 g/L	25 °C; pH = 7; 24 h; 150 rpm shaking	11.28 mg/g	[80]
Triclosan	0.300 mg/L	20–60 μm	0.2 g/L	25 °C; 168 h; 180 rpm shaking; DMSO = 0.1%	0.520 mg/g	[81]
Dyes						
Malachite green	10 mg/L	40 μm	1 g/L	25 °C; pH = 6; 60 h; 160 rpm shaking	4.52 mg/g	[82]
Rhodamine	5 mg/L	40 μm	1 g/L	25 °C; pH = 6; 60 h; 160 rpm shaking	1.27 mg/g	[82]
Flame retardants						
Tetrabromobisphenol A	0.100 mg/L	161 μm	0.020 g/unspecified	25 °C; pH = 7.0 ± 0.2; 48 h; 160 rpm shaking	0.04943 mg/g	[83]
Fungicides						
Azoxystrobin	0.100 mg/L	100 μm	1 g/L	25 °C; pH = 5.5–8.5; 24 h; 150 rpm shaking; NaCl = 0–50 mmol/L	0.017 mg/g	[84]
Carbendazim	1 mg/L	150 μm	0.2 g/L	25 °C; 24 h; 180 rpm shaking	1.5 mg/g	[85]
Hexachlorobenzene	0.100 mg/L	49.7–358 µm	0.333 g/L	25 °C; pH = 7.23 ± 0.06; 96 h; 150 rpm shaking	0.260–0.278 mg/g	[86]
Picoxystrobin	0.100 mg/L	100 μm	1 g/L	25 °C; pH = 5.5–8.5; 24 h; 150 rpm shaking; NaCl = 0–50 mmol/L	0.023 mg/g	[84]
Pyraclostrobin	0.100 mg/L	100 μm	1 g/L	25 °C; pH = 5.5–8.5; 24 h; 150 rpm shaking; NaCl = 0–50 mmol/L	0.081 mg/g	[84]
Herbicides						
1,2,3-Trichlorobenzene	0.100 mg/L	49.7–358 µm	0.333 g/L	25 °C; pH = 7.23 ± 0.06; 96 h; 150 rpm shaking	0.228–0.260 mg/g	[86]
Atrazine	0.200 mg/L	5 μm	0.1 g/L	25 °C; pH = 7; 48 h	0.35040 mg/g	[87]
Butachlor	0.012 mg/L	150 μm	0.2 g/L	25 °C; pH = 7; 2 h	0.01365 mg/g	[88]
Florpyrauxifen-benzyl	7 mg/L	150 μm	25 g/L	25 °C, pH = 4; 48 h; 150 rpm shaking	0.242465 mg/g	[89]
Terbuthylazine	0.600 mg/L	<250 μm	2 g/L	20 °C; pH = 6.5 ± 0.2; 48 h; 200 rpm shaking	0.068 ± 0.0033 mg/g (virgin PE–MPs)	[67]
Terbuthylazine	0.600 mg/L	<250 μm	2 g/L	20 °C; pH = 6.5 ± 0.2; 48 h; 200 rpm shaking	0.029 ± 0.0037 mg/g (aged PE–MPs)	[67]
Trifluralin	0.100 mg/L	49.7–358 µm	0.333 g/L	25 °C; pH = 7.23 ± 0.06; 96 h; 150 rpm shaking	0.307–0.333 mg/g	[86]
Insecticides						
Carbofuran	10 mg/L	150 μm	0.2 g/L	25 °C; 24 h; 180 rpm shaking	4.01 mg/g	[85]
Chlorpyrifos	1 mg/L	100–200 μm	0.2 g/L	30 °C; pH = 7; 24 h; 250 rpm shaking	1.12 ± 0.04 mg/g (ultraviolet–B—aged PE–MPs)	[90]
Imidacloprid	0.200 mg/L	5 μm	0.1 g/L	25 °C; pH = 7; 12 h	0.32775 mg/g	[87]
Lambda-cyhalothrin	10 mg/L	5 μm	0.1 g/L	25 °C; 72 h; 180 rpm shaking	34.4 mg/g (virgin PE–MPs)	[91]
Lambda-cyhalothrin	10 mg/L	5 μm	0.1 g/L	25 °C; 72 h; 180 rpm shaking	39.0 mg/g (aged PE–MPs)	[91]
Medicines						
Propranolol (cation)	0.050 mg/L	45–48 µm	0.2 g/L	25 °C; pH = 7; 96 h; 150 rpm shaking	0.0038 mg/g	[76]
Sertraline (cation)	0.050 mg/L	45–48 µm	0.2 g/L	25 °C; pH = 7; 96 h; 150 rpm shaking	0.0187 mg/g	[76]
Other organic contaminants						
1,2,4–Trichlorobenzene	0.100 mg/L	49.7–358 µm	0.333 g/L	25 °C; pH = 7.23 ± 0.06; 96 h; 150 rpm shaking	0.238–0.282 mg/g	[86]
1,3,5–Trichlorobenzene	0.100 mg/L	49.7–358 µm	0.333 g/L	25 °C; pH = 7.23 ± 0.06; 96 h; 150 rpm shaking	0.249–0.260 mg/g	[86]
9–Nitroanthracene	0.500 mg/L	100–150 μm	0.666 g/L	25 °C; pH = 3–11; 48 h; 60 rpm shaking	0.73435 mg/g	[92]
Pentachlorobenzene	0.100 mg/L	49.7–358 µm	0.333 g/L	25 °C; pH = 7.23 ± 0.06; 96 h; 150 rpm shaking	0.292–0.308 mg/g	[86]
**Adsorption of inorganic elements**						
Cadmium (Cd^2+^)	1 mg/L	30–63 μm	1 g/L	25 ± 1 °C; pH = 7; 5 h; 200 rpm shaking; salinity = 0	1.8 mg/g	[93]
Cadmium (Cd^2+^)	10 mg/L	125 μm	1 g/L	25 °C; pH = 5.5; 48 h; 160 rpm shaking	0.495 mg/g	[94]
Chromium (Cr^3+^)	5 mg/L	48 μm	2 g/L	25 °C; pH = 6; 150 rpm shaking	0.86 mg/g	[95]
Chromium (Cr^3+^)	10 mg/L	125 μm	1 g/L	25 °C; pH = 5.5; 48 h; 160 rpm shaking	1.023 mg/g	[94]
Chromium (Cr^6+^)	100 mg/L	177–250 μm	14 g/L	25 °C; pH = 5; 12 h	0.32 mg/g	[96]
Chromium (Cr^6+^)	5 mg/L	~100 μm	1 g/L	25 °C; pH = 2; 48 h; 150 rpm shaking; detergent = 4% (*v*/*v*)	1.00 mg/g (virgin PE–MPs)	[97]
Chromium (Cr^6+^)	5 mg/L	<250 μm	1 g/L	25 °C; pH = 2; 48 h; 150 rpm shaking; detergent = 4% (*v*/*v*)	0.62 mg/g (aged PE–MPs)	[97]
Copper (Cu^2+^)	5 mg/L	149–250 μm	2.5 g/L	26 °C; 150 rpm shaking	0.315 ± 0.087 mg/g	[98]
Copper (Cu^2+^)	10 mg/L	2380–4760 μm (films)	10 g/L	25 °C; pH = 8; 10 h; 200 rpm shaking	0.53064 ± 0.00835 mg/g	[99]
Copper (Cu^2+^)	10 mg/L	125 μm	1 g/L	25 °C; pH = 5.5; 48 h; 160 rpm shaking	0.286 mg/g	[94]
Lead (Pb^2+^)	5 mg/L	<250 μm	2 g/L	20 °C; pH = 6.5 ± 0.2; 48 h; 200 rpm shaking	0.400 mg/g (virgin PE–MPs)	[67]
Lead (Pb^2+^)	5 mg/L	<250 μm	2 g/L	20 °C; pH = 6.5 ± 0.2; 48 h; 200 rpm shaking	1.200 mg/g (aged PE–MPs)	[67]
Lead (Pb^2+^)	2 mg/L	48 μm	1 g/L	25 °C; pH = 5; 24 h; NaNO_3_ = 10 mM	0.0912 mg/g	[100]
Lead (Pb^2+^)	10 mg/L	2380–4760 μm (films)	10 g/L	25 °C; pH = 8; 10 h; 200 rpm shaking	0.66027 ± 0.00319 mg/g	[99]
Lead (Pb^2+^)	10 mg/L	125 μm	1 g/L	25 °C; pH = 5.5; 48 h; 160 rpm shaking	1.013 mg/g	[94]
Nickel (Ni^2+^)	10 mg/L	2380–4760 μm (films)	10 g/L	25 °C; pH = 8; 10 h; 200 rpm shaking	0.0089 ± 0.00891 mg/g	[99]
Nickel (Ni^2+^)	10 mg/L	125 μm	1 g/L	25 °C; pH = 5.5; 48 h; 160 rpm shaking	0.848 mg/g	[94]
Zinc (Zn^2+^)	10 mg/L	2380–4760 μm (films)	10 g/L	25 °C; pH = 8; 10 h; 200 rpm shaking	0.23835 ± 0.00365 mg/g	[99]
Zinc (Zn^2+^)	10 mg/L	125 μm	1 g/L	25 °C; pH = 5.5; 48 h; 160 rpm shaking	0.554 mg/g	[94]

RPM: revolutions per minute.

**Table 3:** Effects of PE–MPs exposure on different animal, plant, and cell models.

Model/Organism	Size [µm]	Shape	Amount/Concentration of PE–MPs	Time of Exposure	Toxic Effect	Source
Adult albino rats	Unspecified	Unspecified	1.5 mg/kg body weight (orally)	Single dose	Reduction in expressions of antioxidant enzymes, increased levels of reactive oxygen species, reduced level of follicle-stimulating hormone, reduced level of luteinizing hormone, reduced level of testosterone, decreased sperm viability, motility, and sperm count, increase in dead sperms number, and sperm structural anomalies	[175]
C57BL/6 J mice	10–20	Fragments	10 μg/day (orally)	4 months	Accumulation in inner ear, hearing loss	[176]
*Caenorhabditis elegans*	322	Microbeads	1 mg/L	45–60 days + ultraviolet irradiation	Increased oxidative stress and related genes expression	[177]
*Daphnia magna*	17.23 ± 3.43–34.43 ± 13.09	Fragments	5 mg/L	21 days	Lower survival rate, reduced algal feeding, reduced body length, and reduced number of offspring compared to PE–MPs beads (39.54 ± 9.74 μm)	[178]
Earthworm *Eisenia andrei*	<3600 (50% <57)	Fragments/Films	0.005–1.0% (*w*/*w*)	28 days	Increase in oxidative stress biomarkers	[179]
Earthworm *Lumbricus terrestris*	<150	Fragments	7–60% dry weight of soil	60 days	Increased mortality, reduced growth rate	[171]
Earthworm *Metaphire guillelmi*	550–1000	Fragments/Films	0.25% (*w*/*w*)	28 days	Alteration of gut microbiota	[180]
Honeybees	100	Unspecified	10^5^ PE–MPs/mL of honey	15 days	Altered microbiota, increased mortality	[172]
ICR mice	40–48	Unspecified	0.125–2 mg/day (orally)	90 days	Altered number of live births, sex ratio, and body weight of the offsprings, tissue damage, and disturbed immune response in the dams	[181]
Male C57BL/6 mice	<20	Unspecified	5 mg/kg/day (intratracheally)	14 days	Pulmonary inflammation	[182]
Male Wistar rats	34–50	Unspecified	1.5 mg/kg body weight	28 days	Increase in kallikrein-3 level, reduction of testosterone, luteinizing hormone, thyroid-stimulating hormone, free-triiodothyronine, and total cholesterol	[183]
Mice	36 and 116	Microbeads	100 μg/g of food	6 weeks	Altered gut morphology and inflammation	[173]
*Perca fluviatilis*	100–180	Unspecified	1 mg/g of dry food	15 days	Oxidative stress (stronger in liver compared to muscle tissue)	[27]
Terrestrial crustacean woodlice *Porcellio scaber*	56.8 ± 37.9	Fragments/Films	1–5% (*w*/*w*)	28 days	Increase in electron transfer system activity	[184]
*Tigriopus japonicus*	10–30	Unspecified	12.5 mg/L	14 days	Detrimental effects on feeding, egestion, reproduction, survival	[185]
*Chlorella pyrenoidosa*	2.42–300	Unspecified (powder)	5–100 mg/L	96 h	Inhibition of photosynthesis, induction of oxidative stress	[186]
*Lycopersicon esculentum* L.	11.15 ± 3.32–59.84 ± 24.88	Unspecified	1 mg/L	14 days	Oxidative damage	[187]
3-phase in vitro simulated digestion coupled with a tri-culture small intestinal epithelium model (Caco–2, HT–29MTX, Raji B cells)	0.1	Pellets	400 μg/mL	1–2 h	Increased fat digestion and absorption, but no effect on cell health or permeability	[165]
RAW264.7 (mouse macrophage cells), THP–1 (human monocytic leukemia cells)	180 (degraded)	Unspecified	40–80 mg/L	24 h	Cell death attributed to lysosomal dysfunction	[188]
RAW264.7 (mouse macrophage cells), THP–1 (human monocytic leukemia cells), epithelial cells A549 (human alveolar adenocarcinoma cells), HaCaT (human keratinocyte cells), Caco–2 (human intestinal epithelial cells)	231.9 (degraded)	Unspecified	2–200 g/L	24 h	Reduced cell viability, cell death induction	[189]

## Data Availability

No new data were created or analyzed in this study. Data sharing is not applicable to this article.
